# Nurr1 performs its anti-inflammatory function by regulating RasGRP1 expression in neuro-inflammation

**DOI:** 10.1038/s41598-020-67549-7

**Published:** 2020-07-01

**Authors:** Mihee Oh, Sun Young Kim, Jung-Eun Gil, Jeong-Su Byun, Dong-Wook Cha, Bonsu Ku, Woonghee Lee, Won-Kon Kim, Kyoung-Jin Oh, Eun-Woo Lee, Kwang-Hee Bae, Sang Chul Lee, Baek-Soo Han

**Affiliations:** 1 Biodefense Research Center, Korea Research Institute of Bioscience and Biotechnology, Daejeon, 34141 Republic of Korea; 2 Metabolic Regulation Research Center, Korea Research Institute of Bioscience and Biotechnology, Daejeon, 34141 Republic of Korea; 3 Disease Target Structure Research Center, Korea Research Institute of Bioscience and Biotechnology, Daejeon, 34141 Republic of Korea; 4 Department of Functional Genomics, University of Science and Technology (UST) of Korea, Daejeon, 34113 Republic of Korea; 5 MODNBIO Inc., Seoul, 08378 Republic of Korea

**Keywords:** Nuclear receptors, Microglia

## Abstract

Nurr1, a transcription factor belonging to the orphan nuclear receptor, has an essential role in the generation and maintenance of dopaminergic neurons and is important in the pathogenesis of Parkinson’ disease (PD). In addition, Nurr1 has a non-neuronal function, and it is especially well known that Nurr1 has an anti-inflammatory function in the Parkinson’s disease model. However, the molecular mechanisms of Nurr1 have not been elucidated. In this study, we describe a novel mechanism of Nurr1 function. To provide new insights into the molecular mechanisms of Nurr1 in the inflammatory response, we performed Chromatin immunoprecipitation sequencing (ChIP-Seq) on LPS-induced inflammation in BV2 cells and finally identified the *RasGRP1* gene as a novel target of Nurr1. Here, we show that Nurr1 directly binds to the *RasGRP1* intron to regulate its expression. Moreover, we also identified that RasGRP1 regulates the Ras-Raf-MEK-ERK signaling cascade in LPS-induced inflammation signaling. Finally, we conclude that RasGRP1 is a novel regulator of Nurr1’s mediated inflammation signaling.

## Introduction

Nurr1 (NR4A2) belongs to the nuclear receptor (NR)4 family of orphan nuclear receptors^1^. NRs are ligand inducible transcription factors that bind to DNA and regulate the expression of target genes^2^. Developing mesencephalic dopaminergic cells deficient in Nurr1 are unable to express tyrosine hydroxylase^3,4^. Nurr1 deficiency in embryonic ventral midbrain cells causes them not to migrate normally, and they fail to innervate their striatal target areas^5^. It has been suggested that the absence of Nurr1 may be a contributing factor in the pathogenesis of PD^6^. Recently, two human mutations located in exon 1 of the *Nurr1* gene were shown to result in a decreased expression of *Nurr1* mRNA and were associated with familial PD^7^. Additionally, recent studies have proposed that Nurr1 overexpression or modification of Nurr1 expression in stem cells (and neural stem cells) may have an impact on the future of cell therapy for PD^8–11^. It is further supported by a link between altered Nurr1 expression and PD indicating that Nurr1 may have a protection role.

Saijo et al. reported that Nurr1 protects dopaminergic neurons from inflammation-induced neurotoxicity through the inhibition of pro-inflammatory mediator expression in microglia and astrocytes^12^. Nurr1 functions as a key component of a negative feedback loop in both microglia and astrocytes by recruiting CoREST corepressor complexes to NF-κB target genes^12^. They found that a reduction of Nurr1 expression in itself does not affect the death of TH^+^ dopaminergic neurons, but the expression of inflammatory mediators are enhanced, and the survival rate of TH^+^ neurons are decreased in response to inflammatory stimuli in the Nurr1 deficiency condition^12^. Moreover, they mentioned that astrocytes can act as amplifying agents of microglia-derived pro-inflammatory mediators in the production of neurotoxic factors^12–14^. Collectively, the expression of LPS-induced pro-inflammatory genes in microglia lead to paracrine activation of astrocytes^12,13^. Sequentially, this activation accelerates the production of toxic mediators by astrocytes^12,13,15^. They suggested that these toxic mediators have an additive or synergistic effect with neurotoxic factors produced by microglia and ultimately lead to damaged dopaminergic neurons^12–15^. Based on these results, they proposed that Nurr1 suppresses the production of the microglia-derived pro-inflammatory mediators in response to inflammatory stimuli, and the toxic signal is not delivered to astrocytes. Thus, eventually, neuronal cell death can be prevented.

Besides its role in the brain, Nurr1 is also expressed in non-neuronal cell types and has critical roles. In macrophages, the expression level of Nurr1 mRNA is increased by inflammatory stimuli, including LPS^16,17^. For instance, Nurr1 prevents expression of inflammatory genes in human macrophages that are involved in the development of arthrosclerosis^18^. Additionally, Nurr1 is down-regulated compared to healthy controls in both CD14 + monocytes and CD4 + T cells of Multiple sclerosis (MS) patients^19^. Moreover, NURR1 gene expression is also decreased in the peripheral blood lymphocytes (PBL) of Chinese patients with PD compared with controls^20^. They mentioned that lower levels of NURR1 gene expression is significantly associated with an increased risk of PD in males and older subjects, respectively^20^.

Intriguingly, reported observations suggest that reduction of the inflammation response by Nurr1 activation decreases PD related symptoms and DA neuronal loss in both in vivo and in vitro PD models. Kim et al. found that Nurr1 agonist treatment enhances behavioral deficits in a rat model of PD^21^, and Gaynor et al. revealed that a Nurr1 agonist has neuroprotective and anti-inflammatory roles in a rat model of inflammation exacerbated by oxidative damage from 6-OHDA^22^. In addition, Na chang et al. reported that Nurr1 overexpression exerts neuroprotective and anti-inflammatory roles via down-regulating CCL2 in both in vivo and in vitro PD models^23^, and Jodeiri et al. found that Nurr1 agonists protect cells against MPP + induced toxicity through anti-inflammatory and anti-mitochondrial impairment^24^. However, the molecular mechanism by which Nurr1 controls transcriptional repression or activation of inflammatory responses and the potential function of Nurr1 in the inflammatory machinery of PD have not been fully elucidated.

The DNA binding domain of Nurr1 is well-conserved (over 90% homology) among the NR family members and consists of two zinc finger modules^2^. The DNA binding domain of Nurr1 is known to activate transcription by binding to an NGFI-B response element (NBRE)^4,25^. To identify potential novel target genes of Nurr1 in LPS-induced inflammation in BV2 microglial cell lines, we performed chromatin immunoprecipitation sequencing (ChIP seq). Microglial cells are the resident macrophages of the brain^26^. When activated, microglia can be potent immune effector cells^27^. In neurodegenerative disorders such as Alzheimer’s and Parkinson’s disease, microglia are activated and promote the release of pro-inflammatory cytokines to disturb normal CNS activity^28^. The use of primary microglia cultures is widespread in the research of neuroinflammatory reactions due to the resemblance of their phenotype to in vivo cells^28^. However, primary microglia cultures are difficult to use due to the low number of cells and time consuming techniques needed to cultivate primary microglia cultures^28,29^. Thus, methods were developed to get a large number of cells quickly. Such immortalized cell lines can be generated by using a retrovirus^29^. Commonly used cell lines of this type are the BV2 cell lines, which are derived from mice^29^. Actually, BV2 cells have similar functions as primary microglia. Henn et al*.* found that in response to LPS, 90% or more of the genes induced by the BV2 cell lines also are induced by primary microglia^29^. Until now, BV2 cell lines have been widely used in research related to neuro-inflammatory responses. Thus, we choose these cell lines to carry out our experiments.

The identification of genes that are potentially under direct transcriptional control of Nurr1 is important to obtain a more detailed view of the Nurr1 downstream signal cascade. In this study, we found that RasGRP1 is a new player in inflammation signaling mediated by Nurr1 because it is a target site for the DNA binding domain of Nurr1.

The purpose of this study was to elucidate the new mechanisms of the actions of Nurr1. Thus, we attempted to find a novel binding target for the DNA binding domain (DBD) of Nurr1. Herein, we present evidence that RasGRP1 is a negative regulator in inflammation signaling mediated by Nurr1 by binding at its DBD. Nurr1 has a role as a transcriptional repressor of genes that encode inflammatory response factors through RasGRP1. Additionally, we also found that RasGRP1 modulates the Ras-Raf-MEK-ERK signaling cascade in LPS-induced inflammation.

## Methods

### Cell culture and reagents

BV2 cells, a mouse microglial cell line, and human embryonic kidney (HEK) 293 T cells were grown in Dulbecco’s modified eagle’s medium (DMEM) (Invitrogen, Carlsbad, CA, USA) supplemented with 10% fetal bovine serum (FBS) (Invitrogen) at 37 °C in a humidified atmosphere of 95% air and 5% CO_2_. Additionally, BV2 cells stably expressing genes were maintained in DMEM supplemented with 10% FBS and 5 μg/ml puromycin (Invitrogen). BV2 cells were plated at a density of 2 × 10^6^ cells/plate. After 24 h, cells were incubated in 1 μg/ml of lipopolysaccharide (LPS: Sigma-Aldrich, St. Louis, MO, USA) or saline (Welgene Inc., Daegu, Korea) for the indicated time.

### Primary microglial cell culture

Primary microglial cultures were prepared from mixed glial cultures as previously described^30–33^. Briefly, whole brains of newborn pups were mechanically disrupted by chopping, and big fragments were removed using a 215 µM nylon mesh. Then, the filtered pieces were treated with trypsin and DNase (Roche, Penzberg, Germany). The cells obtained were seeded in culture flasks and grown at 37 °C in a 5% CO_2_ atmosphere in DMEM (Invitrogen) supplemented with 10% FBS (Invitrogen). Culture media were changed every 3 days. The cells were cultured for 21 days. Primary microglia were separated from the mixed cultures by vigorously tapping the flasks on the bench top. The floating cells in the conditioned culture media were collected. The resulting cells were purified microglia. The floating cells were counted and seeded onto 10 μg/ml Poly d-Lysine (PDL: Sigma-Aldrich) coated culture dishes.

All experimental protocols were approved by the Institutional Animal Care and Use Committee of the Korea Research Institute of Bioscience & Biotechnology (KRIBB), and were carried out in accordance with the guidelines for the Care and Use of Laboratory Animals published by the US National Institutes of Health.

### Plasmid construction

To construct BV2 cells that stably expressed a GFP-tagged wild type Nurr1 protein, Nurr1 cDNA was amplified by PCR using specific primers that included the sequence for the BamHI restriction site and the cDNA sequence from normal BV2 cells as the DNA template. Then, the fragment was cloned into pGEM T easy vector (Promega, Madison, WI, USA) , confirmed by DNA sequencing and ligated into pLVX-EF1alpha-AcGFP1-C1 (Clontech Laboratories, Mountain View, CA) digested with the same restriction enzymes. To produce recombinant protein, the DBD and LBD of Nurr1 were amplified by PCR using specific primers that included the sequence for the BamHI restriction site and the cDNA from the Nurr1 cDNA cells as the DNA template. Then, the fragment was cloned into pGEM T easy vector (Promega), which was confirmed by DNA sequencing, and then ligated into pGEX 4 T-1 (Amersham Biosciences, Piscataway, NJ, USA), which was digested with the same restriction enzymes. For the luciferase assay, fragment primers for − 850 were designed to amplify the mouse *RasGRP1* promoter. The promoter construct was generated by PCR using specific primers that included the sequences for the KpnI and Xho1 restriction sites and the genomic DNA from normal BV2 cells as the DNA template. Then, the fragment was cloned into pGEM T easy vector (Promega), confirmed by DNA sequencing, and ligated into the luciferase reporter construct, which was a pGL3-basic vector digested with the same restriction enzymes KpnI and Xho1 (NEB). This plasmid was named pGL3-*RasGRP1* 1 kb promoter. And the region containing the consensus elements for Nurr1 (80 base pair) in the *RasGRP1* gene was amplified by PCR using specific primers; the fragment was cloned into a pGEM T easy vector (Promega) and confirmed by DNA sequencing. And then, 4 copies of repeats of the NBRE containing sequence were ligated into the pGL3-*RasGRP1* 1 kb promoter. This plasmid was named pGL3-*RasGRP1* 1 kb promoter + 4X NBRE. The PCR amplification conditions were similar to the conditions used in the previous step.

### Lentiviral particle production

The lentivirus particles used in this study, including GFP, GFP-Nurr1, scrambled RNA, shNurr1 RNA, and shRasGRP1 RNA were generated in HEK 293 T cells. Briefly, a mixture of 3 μg of envelope plasmid (pMD2G), 6 μg of packaging plasmid (psPAX2) and 12 μg of transfer vector was prepared in opti-MEM media (Invitrogen) and transfected into 293 T cells (5 × 10^6^ cells/100 mm dishes) using Lipofectamine 2000 (Invitrogen) and cultured for 48 h. Lentivirus containing supernatant was harvested and passed through a 0.45 μm filter. The supernatant was spun at 13,893×*g* for 90 min to concentrate the pellet, which was resuspended in 150 μl 1 × PBS.

### Cell line preparation

To construct the BV2 cells that stably expressed the GFP-tagged wild type Nurr1 protein, a lentiviral infection system was used. For the expression of Nurr1, virus particles expressing the GFP-tagged Nurr1 was transduced into the BV2 cells. Ectopic expression of GFP-tagged Nurr1 was confirmed by qRT-PCR and western blot analysis.

From Sigma Mission, we purchased a sequence verified viral vector (pLKO.1-puro) based short hairpin RNA library (five clones targeting different sequences in the coding regions of the Nurr1 gene) against Nurr1 (Nurr1-shRNA). The sequence-verified shRNA lentiviral plasmid vectors for the mouse Nurr1 gene were cloned into the pLKO.1-puro vector. One (we encoded it as sh-3) out of five clones appeared to be efficiently knocking down Nurr1 expression. The plasmids contained the following sequences.

- sh-3: CCGGCGATTTCTTAACTCCAGAGTTCTCGAGAACTCTGGAGTTAAGAAATCGTTTTT.

For the control transfections, we purchased the MISSION Non-Target shRNA control vector, a lentivirus plasmid vector. The vector contains the following shRNA insert, CCGGCAACAAGATGAAGAGCACCAACTCGAGTTGGTGCTCTTCATCTTGTTGTTTTT, which does not target any human or mouse gene, making it useful as a negative control in the experiments. And these shRNA constructs were used to produce lentiviruses. To construct the BV2 cells that stably knocked down Nurr1, lentivirus particles encoding the Nurr1 shRNA was transduced into the BV2 cells, and the cells were selected with puromycin (5 μg/ml) treatment over 14 days. At that time, total RNA was isolated to detect the relative mRNA level of the target genes including Nurr1. They were also confirmed by western blot analysis. To construct the BV2 cells that stably knocked down RasGRP1, the same method was adapted, and the plasmids contained the following sequences.

- sh-2: CCGGGCCCTTTACAATCATATCAATCTCGAGATTGATATGATTGTAAAGGGCTTTTTG.
- sh-3: CGGAGAACAAAGAGTCCCTTATAACTCGAGTTATAAGGGACTCTTTGTTCTTTTTTTG.

To construct primary microglia cells that expressed the GFP-tagged wild type Nurr1 protein, a lentiviral infection system was used. After preparation of the primary microglia cells for 1 day, to express Nurr1, virus particles expressing the GFP-tagged Nurr1 was transduced into the primary microglial cells. Then, after 4 day, ectopic expression of the GFP-tagged Nurr1 was confirmed by western blot analysis.

### RNA extraction, reverse transcription, and real-time PCR

To assess mRNA expression, cells were harvested, and total RNA was isolated using the TRIzol reagent (Invitrogen) followed by chloroform mediated phase separation. RNA was precipitated by adding 100% isopropanol and washed with 75% ethanol. The RNA was examined for purity, and the concentration was measured using a Nano Drop spectrophotometer. First-strand complementary DNA (cDNA) synthesis was performed using 2 μg of the total RNA and 0.5 μg of oligo(dT) primer using M-MLV reverse transcriptase (Promega) in according to the manufacturer’s instructions. All quantitative PCR reactions were performed with SYBR Premix Ex Taq™ (Takara Bio Inc., Otsu, Japan) using a CFX96TM Real-Time System (Bio-Rad Laboratories, Hercules, CA, USA). After 95 °C for 5 min, the experimental reaction consisted of 40 cycles of 95 °C for 15 s. and 60 °C for 30 s. The mRNA expression levels were determined by the 2-delta Ct method. The mouse actin gene was used as an internal control.

The primers used were as follows: *IL-1β* (5′-TCAGGCAGGCAGTATCACTC-3′ 5′-AGGATGGGCTCTTCTTCAAA-3′), *IL-6* (5′-AGTCCGGAGAGGAGACTTCA-3′ 5′- ATTTCCACGATTTCCCAGAG-3′), *TNFα* (5′-ATGAGAAGTTCCCAAATGGC-3′ 5′-CTCCACTTGGTGGTTTGCTA3′), *Nurr1* (5′-ATCTCCTGACCGGCTCTATG-3′ 5′-TGGGTTGGACCTGTATGCTA-3′), *RasGRP1* (5′-CCATCTCCAGGCTTAAGGAA-3′ 5′-GGAGGACAGCAGTTCAGTCA-3′) and *Actin* (5′-CATCCGTAAAGACCTCTATG-3′ 5′-ATGGAGCCACCGATCCACA-3′) for the mouse BV2 sample. IL-1β (5′-ACTCATTGTGGCTGTGGAGA-3′ 5′-TAGCAGGTCGTCATCATCCC-3′), IL-6 (5′-TCTCCGCAAGAGACTTCCAG-3′ 5′-TTCTGACAGTGCATCATCGCT-3′), TNFα (5′-TCAGTTCCATGGCCCAGAC-3′ 5′-GTTGTCTTTGAGATCCATGCCATT-3′), RasGRP1 (5′-AGATAGGGAGGGCCTCATCA-3′ 5′-GGGCTTCAGGTAAGTGGTCT-3′), IBA1 (5′-ATGTCCTTGAAGCGAATGCT-3′ 5′-TTCTCAAGATGGCAGATCTCTT-3′) and GAPDH (5′-GGCACAGTCAAGGCTGAGAATG-3′ 5′-ATGGTGGTGAAGACGCCAGTA-3′) primers were used for the rat primary microglia samples.

### Western blotting

The cells were washed with cold phosphate buffered saline (PBS) and lysed using an ice-cold Nonidet-P40 lysis buffer [1% NP-40, 150 mM NaCl, 10% glycerol, 1 mM EDTA, and 20 mM Tris–HCl (pH 7.4)] supplemented with a protease inhibitor cocktail (Roche) and a phosphatase inhibitor cocktail (Roche). Samples were centrifuged for 30 min at 14,000 rpm. Protein concentrations were measured from the supernatants using the Bradford protein assay kit (Biorad). Lysates were run on 10% acrylamide Bis–Tris gels and transferred to PVDF membranes (Millipore, Billerica, MA, USA). After the membranes were blocked in 5% skim milk, 0.05% Tween 20 and Tris-buffered saline (TBS) for 0.5 h, they were incubated with primary antibodies for 12 h on a rocking platform at 4 °C. Specific primary antibodies were anti-Nurr1 (Santa Cruz, 1:500), anti-RasGRP1 (Santa Cruz, 1:500), anti-IL-1β (Cell signaling technology, Danvers, MA, USA, 1:1,000), anti-Ras (Cell signaling technology, 1:200), anti-phospho Raf (Cell signaling technology, 1:1,000), anti-Raf (Abcam, Cambridge, UK, 1:1,000), anti-phospho ERK1/2 (Cell signaling technology, 1:1,000), anti-ERK1/2 (Cell signaling technology, 1:1,000), anti-phospho MEK1/2 (Cell signaling technology, 1:1,000), anti-MEK1/2 (Cell signaling technology, 1:1,000), anti- IBA1 (Abcam, 1:1,000), anti-β-Actin (Sigma-Aldrich, 1:20,000), and anti-α-Tubulin (Sigma-Aldrich, 1:20,000). The membrane was then incubated with 5% skim milk in TBS-T buffer containing HRP-conjugated secondary antibody (Santa Cruz) for 1 h. HRP-conjugated IgG bound to each protein band was visualized using the enhanced chemiluminescence (ECL) method (Millipore). Membranes probed for β-actin were used as an internal control. The protein bands were quantified using image analysis software (Image J, V.1.42, National Institutes of Health, Bethesda, MD), and the protein levels were expressed as a percent (%) of the controls.

### ChIP sequencing

For the in vitro ChIP sequencing, 10 μg of His tagged Nurr1 vector were transfected, and the cells were cultured for 24 h. Five 100 mm dishes were used for each antibody. The protein-DNA complexes were cross-linked with 1% formaldehyde for 10 min and stopped by the addition of glycine to 0.125 M. The cells were washed with cold PBS and lysed in 1 ml of 1 × lysis buffer, pH 8.1 and a protease inhibitor cocktail (Roche). After digesting the DNA with nuclease, the DNA was sonicated to an average length of 100 ~ 500 bp on a sonicator through 15 pulses each 10 s long at one-third of the maximum power. The fragmented chromatin samples were suspended in the ChIP dilution buffer, and the fragmentation status of the chromatin samples was measured by separating on a 2% agarose gel. Chromatin IP was performed with the His antibody or normal rabbit IgG antibody (Ctrl). The ChIP-DNA was elucidated, and after that, the analysis and experiment were performed by Genomictree (Daejeon, South Korea)^34–40^. Briefly, the DNA-end was repaired to overhang a 3′-dA, and then, adapters were ligated to the end of the DNA fragments. The DNA fragments with the proper size (usually 100–300 bp including the adaptor sequence) were selected after PCR amplification. Finally, we got a qualified library for sequencing according to the manufacturer’s instructions (Genomictree). Statistical analysis was performed using Student’s t-test to compare the relative values of enrichment of 5 folds to the relative values of a non-enriched region (control), as determined by the ChIP-on-chip.

### Luciferase reporter assay

The binding capabilities of Nurr1 onto the *RasGRP1* intronic region were evaluated using the Dual-Luciferase expression assay (Promega) according to the manufacturer’s instructions. For luciferase reporter gene analyses, the R*asGRP1* core promoter followed by 850 bases spanning the Nurr1 binding site in the second intron was cloned into the upstream region of the luciferase gene in the pGL3 basic vector. Transfections were performed in 24-well plates with the reporter plasmid by itself (250 ng) or in combination with different amounts of the Nurr1 expression vector (50, 100, 200, or 250 ng) using Lipofectamine 2000 (Invitrogen). Then, 24 h after the transfection, the cells were harvested and lysed using passive lysis buffer (Promega), and then, the Firefly and Renilla luciferase activities were measured with a microplate luminometer according to the manufacturer’s instructions.

### Protein production and GST purification

The pGEX plasmid containing the coding sequence for the GST-tagged construct was transformed into the *Escherichia coli* BL21 strain. Next, 10 ml of Luria–Bertani broth (with 50 μg/ml ampicillin) were inoculated with a single colony and incubated at 37 °C and 220 rpm. Isopropyl-ß-d-thiogalactopyranoside (IPTG) was added to a final concentration of 0.1 mM to induce expression in 200 ml LB media; cells were incubated and cultured overnight at 20 °C for 16 h with 0.1 mM IPTG to avoid aggregation. After the protein expression, the cells were collected by centrifugation and purified by GST resin according to the manufacturer’s instructions. The protein concentration was analyzed by SDS-PAGE (10% gel).

### Electrophoretic mobility shift assay

The concentration of the Nurr1 fusion protein was measured by the Bradford dye assay (Bio-Rad). The LightShift Chemiluminescent EMSA Kit (Thermo Fisher Corp., Waltham, MA, USA) was used for the EMSA according to the manufacturer’s instructions. Briefly, biotin labelled probes were incubated at room temperature for 20 min with a reaction mixture containing 2 μl of 10 × binding buffer, 1 μl of poly (dI^.^dC), and 3.75 μg of protein extract in a final volume of 20 μl. Finally, the protein-DNA complexes were separated on neutral 6% TBE/polyacrylamide gels using 0.5 × TBE buffer for 1.5 h. Competition binding assays were assessed by adding non labelled competitor oligonucleotides (2000 ng) in a molar excess prior to adding the labeled oligonucleotides (25 ng). The gel was transferred to nylon membranes (Millipore, Billerica, MA, USA) at 80 V for 60 min. After the transfer, the membrane was crosslinked for 10–15 min with the membrane face down on a transilluminator. Then, the membrane was washed using a 1 × wash solution, incubated with the substrate, placed in a film cassette, and exposed to the X-ray film for 5–30 s.

### ChIP assay

The ChIP assays were performed using the SimpleChIP® Enzymatic Chromatin IP Kit (Cell signaling technology) according to the manufacturer’s instructions. The samples (n = 3) from the BV2 cells were used. In total, 1 × 10^7^ post-confluent BV2 cells were used for these experiments. The protein-DNA complexes were cross-linked with 1% formaldehyde for 15 min. and stopped by the addition of glycine to a final concentration of 0.125 M. The cells were washed with cold PBS and lysed in 1 ml of 1 × lysis buffer, pH 8.1 containing a protease inhibitor cocktail (Roche). After digesting the DNA with micrococcal nuclease and sonicating it into fragments of approximately 100–500 bp in length, the fragmented chromatin samples were suspended in a dilution buffer. The chromatin samples were immunoprecipitated with 5 μg of the Nurr1 antibody and normal rabbit IgG overnight at 4 °C. The immunoprecipitated complexes were then pulled out of the sample using protein A/G-coupled agarose beads, and the bound chromatin was collected and washed with salt. The eluted ChIP in the Elution Buffer was then treated with proteinase K to digest the proteins, including nucleases, and purified for PCR analysis. We used the immunoprecipitated products from the normal rabbit IgG group as a negative ChIP control.

Primers used were as follows: (5′-GACATTGGCTTAGTCTCT-3′, 5′-TGACCAGCATCTTGAAGA-3′).

### Cas9/CRISPR mediated genome editing

Guide oligonucleotides containing a T7 RNA polymerase binding site were annealed. T7 polymerase was used for in vitro transcription sgRNA synthesis. gRNA and Cas9 protein were co-transfected into BV2 cells. The gRNA detects the endogenous genomic target site and can base pair via its target binding site, recruiting Cas9 protein to generate a double-strand break at the protospacer adjacent motif (PAM) next to the target site. After 3 days, the cells were split, and single colony isolation was assessed. And then, genomic DNA was extracted from each colony, and indel analysis was performed by PCR. PCR products were separated on 2% agarose gel and imaged with the GelDoc™ imager (Bio-Rad).

### Ras activation assay (Ras pulldown assay)

Activation of Ras, GTP-loaded Ras (RasGTP), was analyzed using the glutathione S-transferase-tagged Ras-binding domain of Raf-1 as a probe in a Ras-GTP pulldown assay. For the assay, cells were treated with LPS (1 μg/ml) in a 100 mm dish at 37 °C for 40 min. The cells were harvested and lysed with ice-cold 1 × Lysis buffer (Cell signaling technology) for the pulldowns. The lysates were vortexed and incubated on ice for 20 min followed by centrifugation at 14,000 rpm for 30 min at 4 °C. Five per cent of the lysate was used for the whole-cell lysate, and 95% (800 μg) was used for the pulldowns according to the manufacturer's instructions (Cell signaling technology). The affinity complexes were washed with 1 × lysis buffer and then resuspended in 2 × Laemmli buffer, boiled, and loaded on 12% acrylamide gels. The proteins were separated and transferred to PVDF membranes (Millipore). Then, the membranes were blocked in 5% skim milk, 0.05% Tween 20, and Tris-buffered saline (TBS) for 0.5 h. The membranes were incubated with the Ras monoclonal primary antibody for 12 h on a rocking platform at 4 °C. The membrane was then incubated with 5% skim milk in TBS-T buffer containing HRP-conjugated secondary antibody (Santa Cruz) for 1 h. HRP-conjugated Mouse IgG bound to each protein band was visualized using the enhanced chemiluminescence (ECL) method (Millipore). Membranes probed for total Ras were used as an internal control.

## Results

### Nurr1 suppressed the expression of proinflammatory cytokine genes

**Figure 1:**
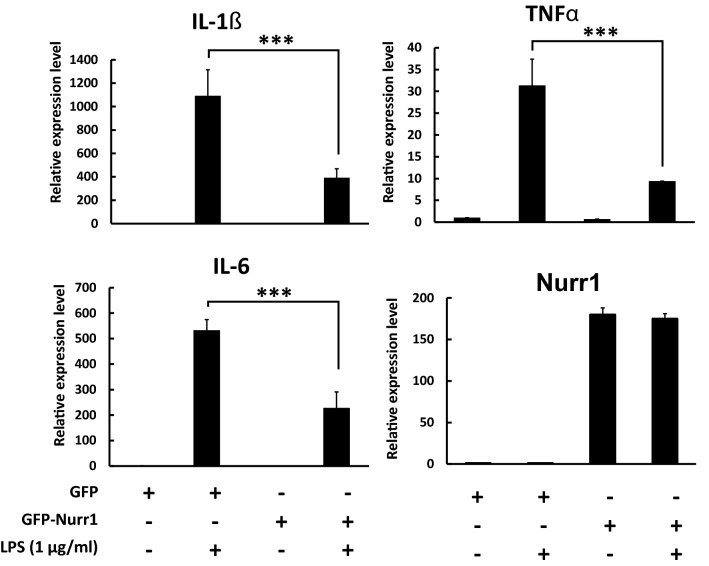
Nurr1 suppressed the expression of proinflammatory cytokine genes. BV2 cells were plated at a density of 2 × 10^6^ cells/plate. After 24 h, cells were incubated in LPS (1 μg/ml) or saline for 6 h. Expression levels of mRNA were analyzed by a semi-quantitative real-time PCR and normalized with actin. This experiment was repeated three times in triplicate using independently prepared mRNAs. Statistical analysis was performed using Student's t-test (mean ± SD; n = 3; ***P < 0.005).

First, we generated BV2 cells expressing Nurr1 using a lentiviral transduction system and fluorescence-activated cell sorting (used to generate a stable cell line) (Supplementary Fig. S1A). Expression of the endogenous Nurr1 protein level was dramatically increased in the Nurr1 over-expressed BV2 cells compared to the control vector, and this data were analyzed by qRT-PCR and western blotting assays (Supplementary Fig. S1A, B). Because it was reported that Nurr1 has an important role in inflammation in microglia and astrocytes, we investigated the effects of the over-expressed Nurr1 on the expression of proinflammatory cytokine genes in BV2 cells. When the cells were treated with inflammation-inducing lipopolysaccharide (LPS; 1 μg/ml) for 6 h in a serum free condition, the expression of proinflammatory genes including interleukin-1β (*IL-1β*), interleukin-6 (*IL-6*) and tumor necrosis factor alpha (*TNFa)* were dramatically induced, as expected (Fig. 1). It was also found that the over-expression of Nurr1 prominently reduced the expression of the proinflammatory genes compared with the control shown by the qRT-PCR and western blotting assays (Fig. 1).

### In vitro ChIP sequencing revealed the binding of Nurr1 to specific regions of Nurr1-regulating genes

To obtain a more detailed view of the Nurr1 downstream cascade in inflammation, the identification of genes that are potentially under direct transcriptional control of Nurr1 is important. To identify novel target genes of Nurr1 in LPS-induced inflammation in BV2 cells, we performed the chromatin immunoprecipitation sequencing (ChIP seq) assay. The immunoprecipitation (IP) was performed with His or normal rabbit IgG antibodies, and the IP-efficiency was determined by western blot analysis (Supplementary Fig. S2A). ChIP for exogenous Nurr1-DNA complexes was performed on transiently expressed His tagged Nurr1 in BV2 cells. The Nurr1-DNA complexes were cross-linked with 1% formaldehyde, stopped by the addition of glycine and then harvested. After digesting the DNA with micrococcal nuclease and sonicating into fragments of approximately 100–500 bp in length, the fragmentation status of the chromatin samples was measured by DNA gel electrophoresis (Supplementary Fig. S2B). The concentration and quality of the immunoprecipitated protein-DNA complexes were analyzed by DNA gel electrophoresis (Supplementary Fig. S2C). We found that 46 Nurr1 specifically enriched genes with a fold enrichment of more than 5 times are involved in the inflammation signaling cascade. Well-known genes involved in inflammation, including *IL-4* and *IL-1β*, were found among the 46 most enriched genes. The list of the enriched genes is given in Table 1.

**Table 1:** Identification of a novel Nurr1-interacting DNA.

Gene symbol	Fold enrichment	Gene symbol	Fold enrichment
Il4	38.73	C6	11.39
Il20rb	31.9	Notch1	11.39
Cxcl5	31.9	Vnn1	11.39
Abr	29.15	Mgll	11.39
Rasgrp1	25.06	Naip2	11.12
Trp73	25.06	Ednra	10.5
Serpinf1	22.79	Chi3l3	9.54
Camk1d	22.41	Scn9a	9.27
Pparg	20.51	Agtr1a	9.25
Prkca	20.51	Hc	9.11
Il1b	18.01	Npy5r	9.11
Stat5b	16.69	Cd55	9.11
Naip6	15.95	Camk4	9.11
Tnfrsf1b	15.95	Pla2g4a	9.11
Slit2	15.95	Il1f6	8.91
Cd44	13.67	Calcrl	7.95
Ggt1	13.67	Ins2	7.95
Cela1	12.83	Prkd1	6.84
F8	11.39	Mecom	6.84
Aoah	11.39	Chia	6.84
Jak2	11.39	Chrna7	6.38
Stk39	11.39	Alox5	6.34
Ccr5	11.39	Chi3l4	5.35

High-throughput sequencing with chromatin immuno-precipitation was performed to identify specific Nurr1 protein–DNA interactions. In three sets of experiments, the potential interacting partners of Nurr1 displaying a significant fold enrichment in all sets were described. Statistical analysis was performed using Student's t-test, comparing the relative values of enrichment of 5 folds to the relative values of a non-enriched region (control), as in ChIP sequencing.

### Nurr1 down-regulated *RasGRP1* mRNA and protein following LPS stimulation

**Figure 2:**
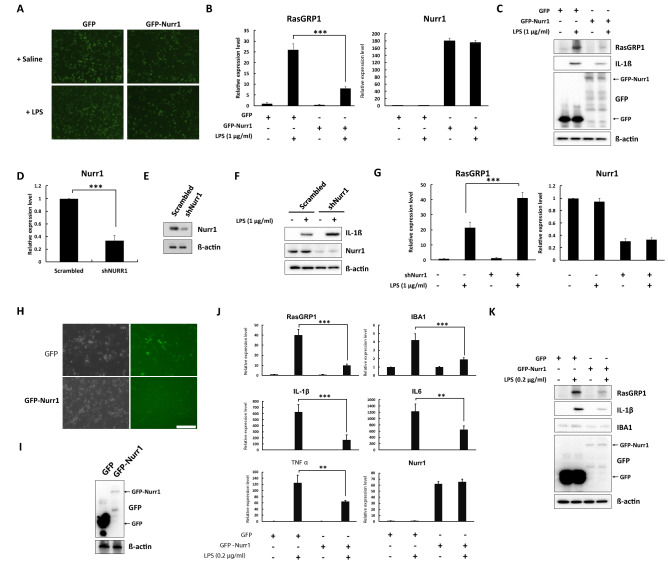
Nurr1 down-regulated *RasGRP1* mRNA and protein following LPS stimulation. (**A**–**C**) BV2 cells expressing GFP or GFP-tagged Nurr1 were treated with LPS (1 μg/ml) or saline for 6 h. (**A**) Images were obtained under a fluorescence microscope. The expression levels of *RasGRP1* mRNA (**B**) and protein (**C**) were analyzed by a semi-quantitative real-time PCR and western blot. (**D**, **E**) BV2 cells were infected with lentiviral particles containing scrambled or shNurr1 RNA. Stable cell lines were selected using puromycin (5 μg/ml). Expression levels of *Nurr1* mRNA (**D**) and protein (**E**) were analyzed using a semi quantitative real-time PCR and western blot. (**F**, **G**) BV2 cells expressing scrambled or shNurr1 RNA were treated with 1 μg/ml LPS for 6 h. (**F**) The expression level of IL-1ß was analyzed by western blot. (**G**) The expression level of *RasGRP1* mRNA was analyzed by semi-quantitative real-time PCR and normalized with actin. (**H**–**K**). Primary microglia cells were infected with lentiviral particles containing GFP or GFP tagged Nurr1. (**H**) Images were obtained under a fluorescence microscope. Scale bars, 500 μm. (**I**) The expression level of Nurr1 was analyzed by western blot. Primary microglia cells expressing GFP or GFP-tagged Nurr1 were treated with LPS (0.2 μg/ml) or saline for 3 h. (**J**) The expression of proinflammatory cytokine genes, *IBA1*, and *RasGRP1* mRNA were analyzed by a semi-quantitative real-time PCR. Additionally, all data were normalized with *GAPDH*. (**K**) The expression level of IL-1ß, IBA1, and RasGRP1 was analyzed by western blot. These experiments were repeated three times. Statistical analysis was performed using Student's t-test (mean ± SD; n = 3; **P < 0.01, ***P < 0.005).

To ensure the reliability and consistency of our data, the results of the array were subsequently validated by qRT-PCR using the BV2 cells (Supplementary Fig. S3). Among several candidates that showed a significant correlation in the ChIP sequencing data, *RasGRP1* was transcriptionally upregulated in the LPS (1 μg/ml) treated BV2 cells for 6 h in a serum free condition (Fig. 2A, B). It was also found that the LPS treated BV2 cells overexpressing Nurr1 prominently had a reduced mRNA expression of *RasGRP1* (Fig. 2A, B). The protein levels were also decreased when compared to the control (Fig. 2C). To verify the data, a shRNA mediated knockdown system was used to reduce the endogenous Nurr1 expression in the BV2 cells. We generated BV2 cells expressing shRNA against Nurr1 (shNurr1) using a lentiviral transduction system and puromycin selection. Expression of the endogenous *Nurr1* mRNA and protein levels was dramatically reduced in the BV2 cells with the Nurr1 knock-down vector compared to those with the scrambled vector (Fig. 2D, E). It was also found that the LPS treatment of the Nurr1 knocked down BV2 cells prominently increased the expression of IL-1β (Fig. 2F). Strikingly, the expression level of *RasGRP1* was increased by shNurr1 (Fig. 2G). We also validated these results in primary microglia cell cultures. Primary microglial cultures were prepared from mixed glial cultures as previously described^30–33^ with minor modifications. We constructed the cells with the lentivirus mediated system for Nurr1 over expression (Fig. 2H, I). Primary microglia cells expressing GFP or GFP-tagged Nurr1 were treated with LPS (0.2 μg/ml) for 3 h to induce the inflammatory response. We analyzed the expression level of the proinflammatory genes including *IL-1β*, *IL-6*, and *TNFa*, microglial cell marker IBA1, and RasGRP1. As a result, we also found that the over-expression of Nurr1 in the inflammation condition reduced the expression of the pro-inflammatory genes, IBA1, and RasGRP1compared with the control shown by the qRT-PCR and western blotting assays (Fig. 2J, K). Based on the data presented here, we identified the existence of a novel regulator at the transcriptional and translational levels, which is crucial for Nurr1’s mediated inflammation signaling in BV2 microglia cell lines and primary microglia cells.

### Nurr1 repressed the transcriptional activity of the *RasGRP1* gene

**Figure 3:**
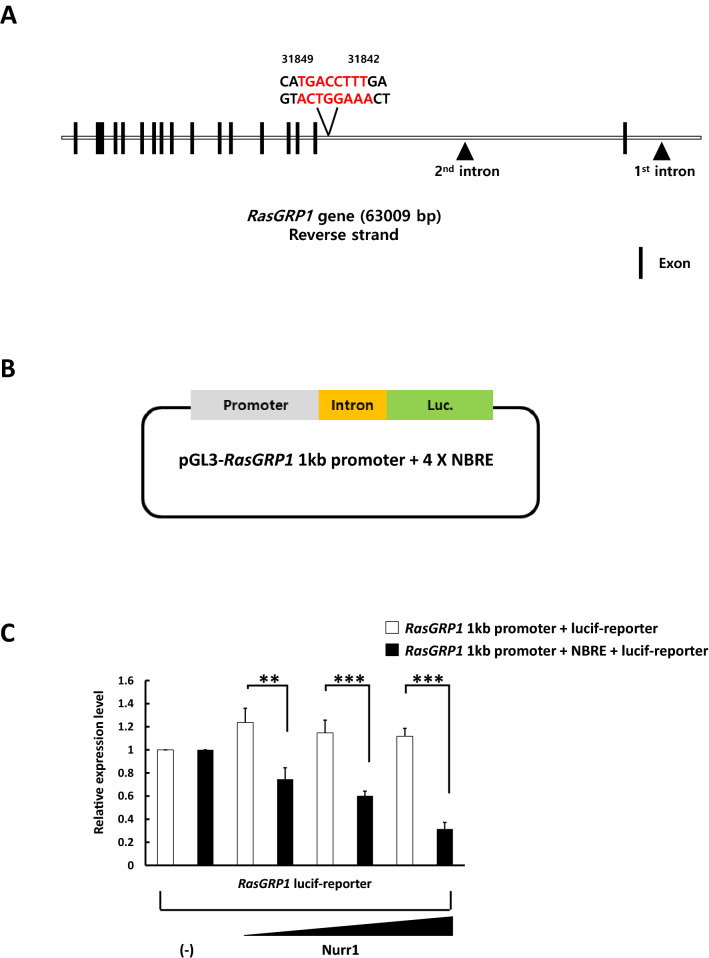
Nurr1 repressed the transcriptional activity of the *RasGRP1* gene. (**A**) Schematic representation of the mouse *RasGRP1* gene, indicating the position of NBRE in the *RasGRP1* intron. The second intron region in the *RasGRP1* gene contains the NBRE (AAAGGTCA) sequence. The *black boxes* represent the exons. (**B**) Schematic diagram of the luciferase reporter construct used in the assays. (**C**) 293 T cells were transfected with either the reporter plasmid alone (250 ng) or a combination of different amounts of the Nurr1 expression vector (50, 100, or 250 ng) using Lipofectamine 2000. After 24 h of transfection, the cells were harvested and lysed, and the luciferase activity was quantified with the dual-luciferase assay system. *Renilla* activity was used for normalization. This experiment was repeated three times using independently prepared cell lysates. Statistical analysis was performed using Student's t-test (mean ± SD; n = 3; **P < 0.01, ***P < 0.005).

The DNA binding domain of Nurr1 is highly conserved between the NR family members. As monomers, Nurr1 binds to the NGFI-B response element (NBRE, 5′-AAAGGTCA-3′)^4^. Thus, we hypothesized that the binding of Nurr1 at the NBRE site in *RasGRP1* would be responsible for the observed changes in the expression level of RasGRP1. Using an in silico analysis for the NBRE sequence in the *RasGRP1* gene, we identified a NBRE at a specific intron site in the *RasGRP1* gene (Fig. 3A). We used a luciferase reporter assay to determine the relevance of the NBRE sequence in the *RasGRP1* gene for the regulation of the Nurr1 mediated inflammation signaling. The *RasGRP1* promoter, followed by an 850 base pair region surrounding the intron site with NBRE, was cloned upstream of the gene encoding the firefly luciferase. The expression of the luciferase gene is thus under the direct control of the *RasGRP1* promoter and the intron region (Fig. 3B). When compared with the empty vector, the *RasGRP1* promoter-reporter vector had significant activity. However, exogenous expression of Nurr1, done by co-transfecting the Nurr1 expressing plasmid along with the reporter vector, reduced the reporter activity. Moreover, the *RasGRP1* promoter-reporter vector had a dose-dependent effect with increasing amounts of the Nurr1-expressing plasmid leading to a significant reduction in the *RasGRP1* reporter activity (Fig. 3C). Thus, we concluded that the NBRE sequence was essential for the Nurr1 mediated regulation of the expression of RasGRP1.

### Nurr1 bound to a specific site in the second intron of the *Rasgrp1* gene

**Figure 4:**
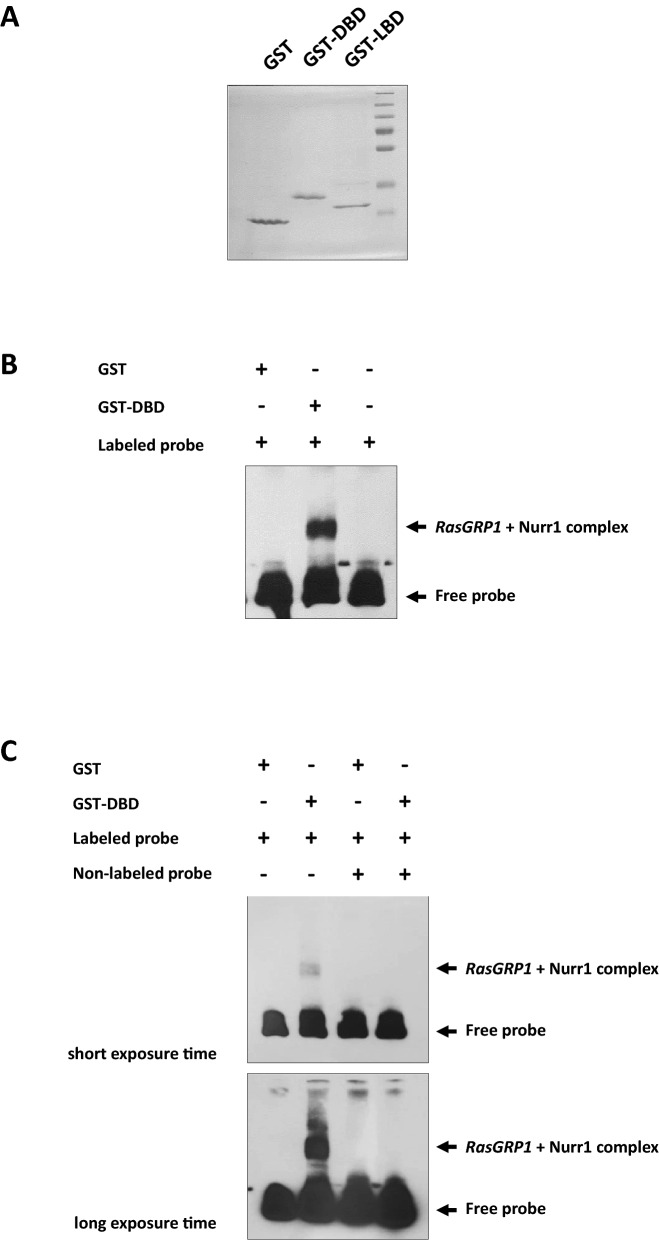
DBD of Nurr1 bound to the NBRE-motif located in the second intron of the *RasGRP1* gene. (**A**) SDS–PAGE gel stained with Coomassie brilliant blue, showing the affinity purification of the recombinant Nurr1 protein used for the electrophoretic mobility shift assay (EMSA). (**B**) EMSA for testing the binding ability of the NBRE-motif containing DNA and the DBD of the purified recombinant Nurr1. Biotin-labeled DNA probe (25 ng) was incubated with purified recombinant GST and GST-DBD (3.75 μg), and the DNA–protein complexes were separated on 6% native polyacrylamide gels. (**C**) The specific binding ability of the DBD of the Nurr1 protein and NBRE-motif containing probe of the *RasGRP1* gene was analyzed by competition assay using non-labeled oligonucleotide (2,000 ng). Non-labeled probe (2000 ng) and biotin-labeled DNA probe (25 ng) from the promoters were incubated with purified recombinant GST and GST-DBD (3.75 μg), and the DNA–protein complexes were separated on 6% native polyacrylamide gels. These experiments were repeated three times.

To correlate the above functional data with the Nurr1-binding properties, we analyzed and compared the binding affinities of the putative regulatory motifs of RasGRP1 to the Nurr1 protein. We assessed the binding of the DBD of Nurr1 to the specific intron site in *RasGRP1* by electrophoretic mobility shift assay (EMSA) using a 150 bp 5′ biotin-labeled DNA probe comprised of the intron site with the NBRE in the *RasGRP1* gene. First, we synthesized the recombinant Nurr1 fusion protein with the DNA binding domain of Nurr1 and GST or the ligand binding domain (LBD) and GST and then expressed these proteins using an *E. coli* expression system followed by purification with a GST purification system. We identified the recombinant Nurr1 fusion protein by electrophoresis on a 12% polyacrylamide gel (PAGE) and then stained it with Coomassie brilliant blue (Fig. 4A). Next, we synthesized a biotin-conjugated DNA oligonucleotide spanning a particular site by PCR amplification, and we finally used it as the probe in the EMSA. In the EMSA, the DBD of Nurr1 generated major DNA–protein complexes when the biotin-conjugated NBRE containing oligonucleotide in the *RasGRP1* gene was used as a probe (Fig. 4B). In addition, the formation of the bands was specifically blocked by a molar excess of unlabeled oligonucleotide (Fig. 4C). Expectedly, the DNA–protein complexes were not clearly detected when the LBD of Nurr1 was used in the EMSA reaction (Supplementary Fig. S4). Thus, these data demonstrate that the DBD of Nurr1 specifically binds to the intron site containing the NBRE sequence in the *RasGRP1* gene.

### Nurr1 is recruited to the second intron in the *RasGRP1* gene

**Figure 5:**
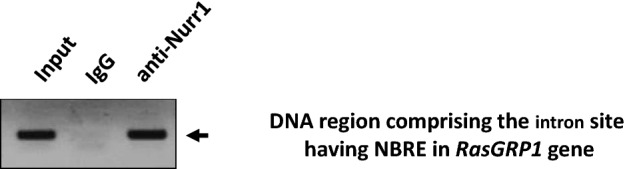
Nurr1 bound to a specific site in the second intron of the *RasGRP1* gene. ChIP assay using an anti-Nurr1 antibody or normal rabbit IgG was performed with BV2 cells, and isolated DNA was subsequently used for PCR with primers flanking the NBRE sequence in the *RasGRP1* gene. PCR specificity was confirmed by agarose gel electrophoresis of the PCR products. This experiment was repeated three times (n = 3).

We did further ChIP assays to confirm the recruitment of Nurr1 to a particular intronic sequence in *RasGRP1*. The ChIP assays were performed in BV2 cells. The DNA protein complexes were cross-linked, harvested, lysed, sonicated, and immunoprecipitated with anti-Nurr1 antibody or a control rabbit IgG. Immunoprecipitated DNA was isolated, and the presence of the specific intron DNA was quantified by PCR analysis. We observed the recruitment of the specific intron region of *RasGRP1* in the anti-Nurr1 antibody sample and not in the control rabbit IgG sample (Fig. 5) confirming the intron site containing that the NBRE sequence in *RasGRP1* is the specific target of Nurr1.

### NBRE containing sequence in the intron of the *RasGRP1* gene has a role in Nurr1 mediated inflammation signaling

**Figure 6:**
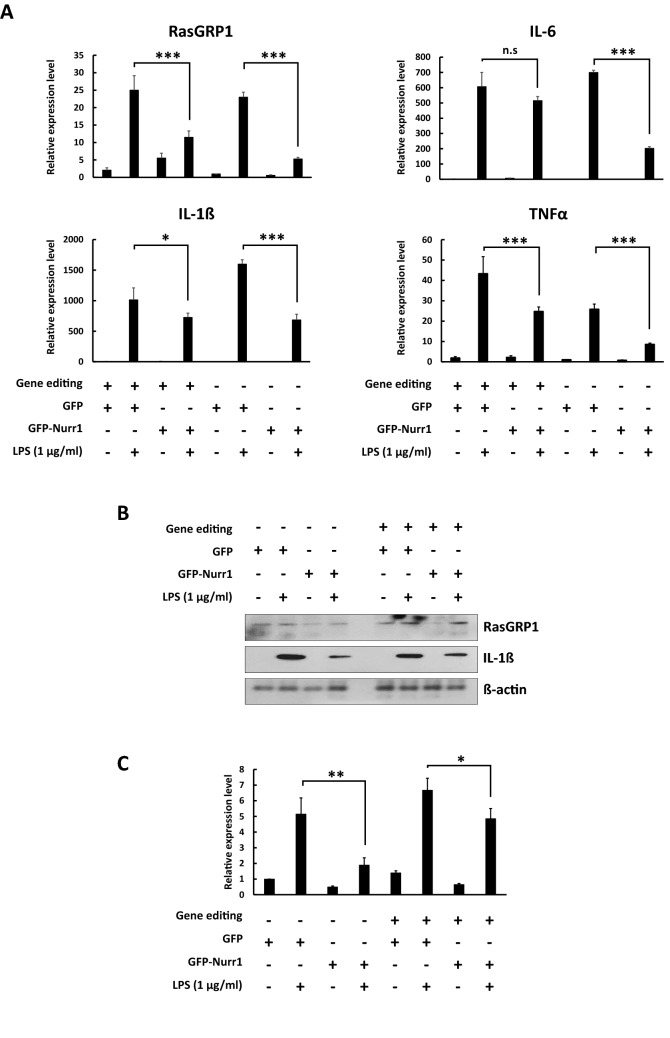
Cas9-mediated deletion of the NBRE sequence in the *RasGRP1* intron partially reduced the LPS-induced expression level of proinflammatory cytokine genes and the *RasGRP1* gene/protein. (**A**–**C**) By Cas9-mediated deletion of the NBRE sequence, Nurr1 partially suppressed the LPS-induced expression level of proinflammatory cytokine genes and *RasGRP1* gene. BV2 cells were treated with LPS (1 μg/ml) for 6 h. The expression level of mRNA (**A**) and protein (**B**) were analyzed by a semi-quantitative real time PCR and western blot. (**C**) The expression level of the RasGRP1 protein was quantitatively analyzed by densitometry. These experiments were repeated three times. Statistical analysis was performed using Student's t-test (mean ± SD; n = 3; *P < 0.05, **P < 0.01, ***P < 0.005).

To further investigate the above functional data about the binding properties of Nurr1 in the *RasGRP1* gene, we did Cas9 mediated gene editing, which deleted the NBRE containing intron sequence in the *RasGRP1* gene, which was confirmed by the Indel analysis. Next, we analyzed the expression level of *RasGRP1*, *IL-1β*, *IL-6*, and *TNFα* in BV2 cells treated for 6 h with LPS (1 μg/ml) stably expressing GFP or GFP-Nurr1 with the Cas9-mediated gene edit. We found that the expression levels of *RasGRP1*, *IL-1β*, *IL-6*, and *TNFα* were more slightly decreased in the cells with the specific intron sequence deleted in the *RasGRP1* gene compared with the control (Fig. 6A). Additionally, we also found that the expression level of RasGRP1 was decreased in the cells that had the specific intron sequence deleted in the *RasGRP1* gene compared with the control (Fig. 6B, C). We demonstrated that the NBRE containing intron sequence in the *RasGRP1* gene has a role in Nurr1 mediated inflammation signaling.

### Ras activation was decreased in the RasGRP1 knockdown cell lines

**Figure 7:**
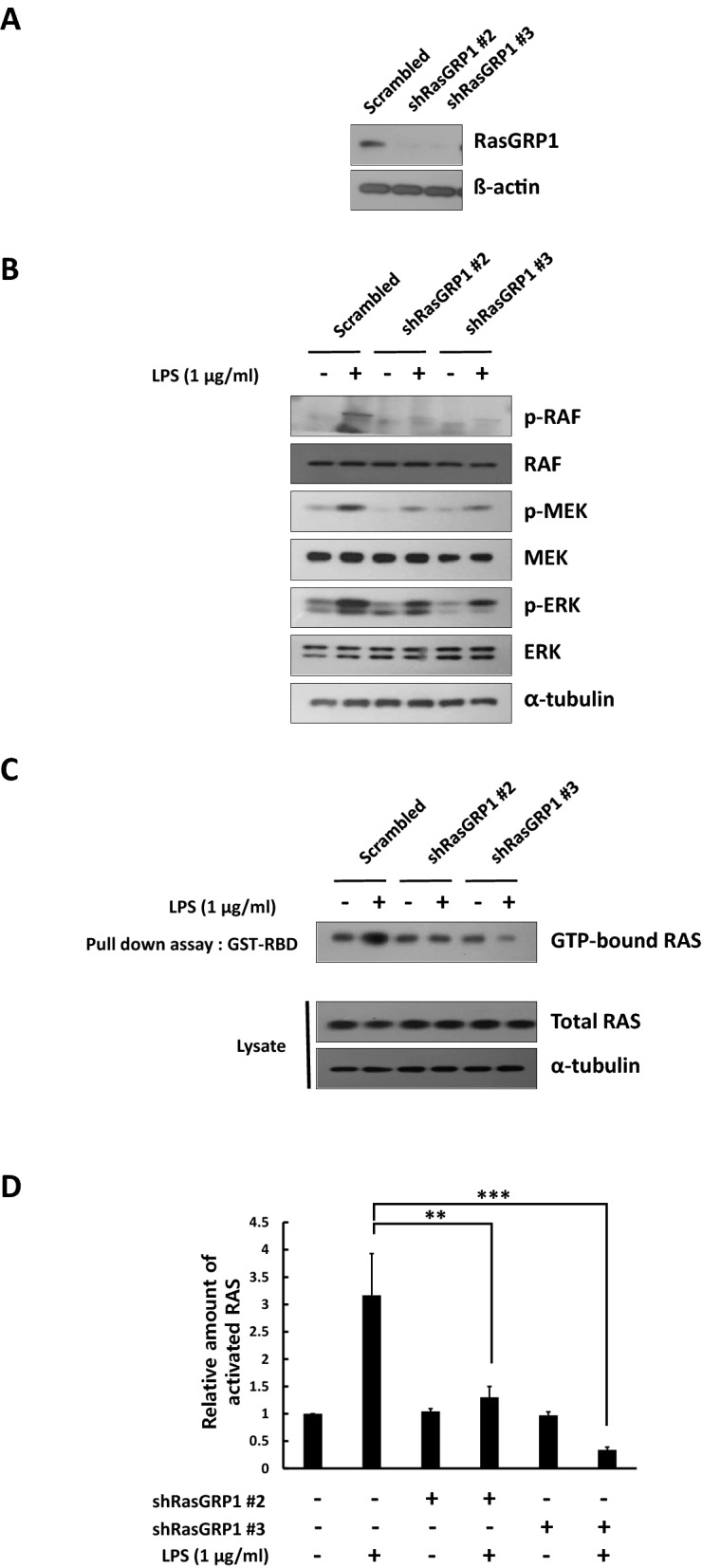
Ras activation was decreased in the RasGRP1 knocked down cell lines treated with LPS. (**A**) BV2 cells were infected with lentiviral particles containing scrambled or shRasGRP1 RNA. Infected cells were selected by puromycin treatment (5 μg/ml). The expression level of RasGRP1 was analyzed by western blot. (**B**) BV2 cells expressing scrambled or shRasGRP1 RNA were treated with LPS (1 μg/ml) for 40 min. The expression level of the downstream signal cascade of RasGRP1 was analyzed by western blot. (**C**, **D**) BV2 cells expressing scrambled or shRasGRP1 RNA were treated with LPS (1 μg/ml) for 20 min. (**C**) GST-fused Ras binding domain (RBD) was used for the pulldown assay to determine the level of GTP-bound active Ras protein in the cell lysates. A parallel western blot analysis of the total cell lysate was performed to determine the total Ras expression level. (**D**) Data were quantitatively analyzed by densitometry. These experiments were repeated three times. Statistical analysis was performed using Student's t-test (mean ± SD; n = 3; **P < 0.01, ***P < 0.005).

The Ras/Raf/mitogen-activated protein kinase (MEK)/extracellular signal-regulated kinase (ERK) signaling pathway has key roles in the transmission of multiple developmental signals and pathologic systems from membrane-bound receptors^41^. A previous study on T cell lines reported that they have a molecular mechanism for Ras and ERK activation downstream of RasGRP1^42^. Small GTP-binding proteins (or GTPases) are a family of proteins that serve as molecular switches in signaling transduction cascades^43^. The 21 kDa Ras proteins are well known for their regulatory role in a variety of biological response pathways including cell growth, transformation, and tumor invasion^44,45^. Like other small GTPases, Ras is activated by GTP binding while the GDP bound form is inactive. In its active (GTP-bound) state, Ras binds specifically to the Ras-binding domain (RBD) of Raf1 to regulate downstream signaling transduction pathways^43^. To determine the relevance of the signaling cascades of RasGRP1 for the regulation of the Nurr1 mediated LPS induced inflammation, we assessed the Ras activity in a LPS-treated control and in RasGRP1 knocked down BV2 cells. First, we generated BV2 cells expressing shRNA against RasGRP1 (shRasGRP1) using a lentiviral transduction system. Expression of the endogenous RasGRP1 protein level was dramatically reduced in the cells transduced with the shRasGRP1 vector compared to the scrambled vector (Fig. 7A). Prior to beginning the Ras activation assay, we analyzed the expression level of the Ras signal cascade molecules in the LPS treated control and in the RasGRP1 knocked down BV2 cells. We found that the phosphorylation levels of Raf1, MEK1/2, and ERK1/2 were decreased in the RasGRP1 knocked down BV2 cells (Fig. 7B). The activation assay used Raf1-RBD agarose beads to selectively separate and pull-down the active form of Ras from the endogenous cell lysates. Subsequently, the precipitated GTP-Ras was detected by western blot analysis using an anti-Ras monoclonal antibody (Fig. 7C and D). As a result, we found that the knock-down of RasGRP1 in the BV2 cells decreased the expression of active Ras in response to LPS.

## Discussion

Nurr1, a transcription factor belonging to the orphan nuclear receptor, has a crucial role in the generation and maintenance of mature midbrain-type dopaminergic neurons in the brain^1,21^. In a previous report, it was shown that Nurr1 also has anti-inflammatory functions by recruiting CoREST corepressor complexes to NF-kB target genes^12^. In this study, we proposed a novel mechanism for Nurr1 mediated anti-inflammation signaling. We showed, for the first time, that Nurr1 modulated LPS-induced inflammatory signal cascade by binding to a specific site in the second intron of the RasGRP1 gene. Nurr1 negatively regulated the RasGRP1 expression, and this could be achieved by the binding of Nurr1 at this particular site. Additionally, we also found that RasGRP1 regulated the Ras-Raf-MEK-ERK signaling cascade in LPS-induced inflammation. Thus, we provide the elucidation of previously unknown regulatory process for the novel factors involved in Nurr1-mediated anti-inflammatory signals.

Commonly, transcriptional modulation involves various factors binding to a promoter or initiation site of a protein coding sequence. Nevertheless, there are numerous studies demonstrating instances in which transcription factor binding to intron regions can regulate the expression of a gene. A previous study reported that SP1/SP3 transcription factors enhance the expression of the UCP3 gene because they bind to an intron of the UCP3 gene^46^. Another report shows that GATA-2, a transcription factor that has important roles in various organs during embryogenesis, is regulated by *Gata2* endothelium enhancer in the fourth intron region, and Gata2 modulation by this enhancer is restricted to the endocardial, lymphatic, and vascular endothelium^47^. Additionally, a recent report shows that Kruppel-like Factor 5 (KLF5), a known transcriptional activator in bladder cancer, binds to a novel enhancer element within the first intron region of CCNE1^48^. Moreover, intron-mediated regulation of gene expression is not restricted to transcriptional enhancement. For instance, Ikaros has been shown to bind to a specific site in the PP2A gene, which represses its expression^49^. Additionally, another study showed that ZEB binds to the first intron of the p73 gene, and its association causes the down regulation of gene expression^50^. In this study, we also showed that Nurr1 binds to a specific intron site in the Rasgrp1 gene and represses its transcriptional and translational expression. The mechanism on how exactly an intron sequence regulates gene expression is still not fully understood. Perhaps the structure of the DNA brings these distal enhancers/repressors closer to the regulatory elements of the promoter of the target genes.

Nurr1 can act as an activator or repressor of transcription, depending on which proteins it binds to and recruits^51^. Repression is affected by a variety of transcription factors and cofactors, which use different pathways to modulate their function. Conventionally, the repressive mechanisms can be modulated by chromatin modification, recruitment of co-repressors with the basal transcriptional machinery, and direct competition between repressors and activators^52–55^. One instance in the case of Ikaros is that it recruits HDAC to a specific site in the intron of the PP2A gene, and its repressive activity is dependent on the presence of HDAC1^48^. Another study shows that the recruitment of Rpd3 histone deacetylase represses transcription activity by inhibiting the recruitment of Swi/Snf, SAGA, and TATA binding protein^54^. In this study, however, we did not find more detailed molecular transcriptional partners in terms of Nurr1 and RasGRP1. Thus, we suggest that additional factors/proteins involved in the regulation of the RasGRP1 remain to be investigated.

Ras is activated through GTP-loading by Ras guanine nucleotide exchange factors (RasGEFs) in response to cell surface receptor signals^56^. The amplitude and duration of epidermal growth factor receptor (EGFR) signaling to Ras and its downstream target MAP kinase (MAPK) affect the fate of the cell; EGF stimulation of rat adrenal pheochromocytoma (PC-12) cells results in transient Ras activation and proliferation whereas nerve growth factor (NGF) stimulation leads to consistent activation of Ras-MAPK, exiting mitosis, and differentiation^57,58^. RasGRP1 is one of the guanine nucleotide exchange factors (GEF) that transduces intracellular signaling to Ras small GTPases. GEFs catalyze the release of GDP by reaching into the nucleotide binding site of small G proteins and opening it, enabling GDP to escape and GTP to bind. RasGRP1 is abundantly expressed in thymocytes and mature T cells but is also expressed in other hematopoietic cells and in the brain. RasGRP1 is activated by membrane translocation, which is driven by binding of its C1 domain to diacylglycerol^59^.

Ras guanine-releasing protein 1 (RasGRP1) is a member of the guanine nucleotide exchange factors to activate Ras proteins^60^. RasGRP1 is crucial for mature T cell signaling, thymocyte differentiation, and B cell proliferation^60,61^. Additionally, RasGRP1 mRNA and protein are highly expressed in the brain^62^. It is found in the olfactory bulb, cortex, caudo-putamen (including striatum), hippocampus, and thalamus. It is strongly up-regulated in a dyskinesia model of PD. A recent study shows that RasGRP1 promotes amphetamine-induced motor behavior through a Ras homolog enriched in the striatum (Rhes) interaction network. That study demonstrated that both amphetamine and RasGRP1 change the expression level of proteins that are associated with various neurological diseases^63^. However, the detailed molecular mechanism of RasGRP1 in the brain remains fully elusive. Additionally, most neurodegenerative conditions are accompanied by neuro-inflammation, yet the exact mechanisms of inflammatory processes are not well understood^64^. Thus, to study whether and how Nurr1 and RasGRP1 are involved in inflammation signaling in the brain may be helpful to elucidate their potential roles in neurological disorders.

In the present study, we addressed whether the binding properties of Nurr1 in the *RasGRP1* gene affect its functions related with Nurr1 mediated inflammation signaling. We used the Cas9 mediated gene editing tool to delete the NBRE containing the intron sequence in the *RasGRP1* gene. We showed that the expression level of *RasGRP1*, *IL-1β*, *IL-6*, and *TNFα* were more slightly decreased in the cells with the specific intron sequence deleted in the *RasGRP1* gene compared with the control. Thus, we demonstrated that the NBRE containing intron sequence in the *RasGRP1* gene has a role in Nurr1-mediated inflammatory signaling. However, we also found that the expression levels of *RasGRP1*, *IL-1β*, *IL-6*, and *TNFα* were not completely suppressed. That means other factors/proteins or pathways participate in the Nurr1 mediated signal cascade because RasGRP1 is not the sole factor in this regulation^65–67^.

Taken together, we suggest that Nurr1 can modulate the expression of RasGRP1 at the transcriptional level and has a role as an anti-inflammatory mediator in neuro-inflammation. By repressing RasGRP1, the expression of inflammatory cytokines was also decreased, and the related signal cascade, the Ras-Raf-MEK-ERK signaling cascade, was partially influenced. Further in vitro and in vivo studies should be performed to find the translational partner for Nurr1 in inflammation.

## Conclusions

In inflammatory conditions induced by LPS, Nurr1 can negatively regulate the RasGRP1 expression at the transcriptional level and has a role as an anti-inflammatory mediator in neuro-inflammation. By repressing RasGRP1, the expression of inflammatory cytokines was decreased, and the related signal cascade, the Ras-Raf-MEK-ERK signaling cascade, was partially influenced.

### Ethics approval and consent to participate

Not applicable.

### Consent for publication

All authors agree to the publication of this manuscript.

## Supplementary information

Supplementary file1 (PDF 1699 kb)

## Data Availability

All data or analyzed during this study are included in this published article.
