# Supplementary material for: Nurr1 performs its anti-inflammatory function by regulating RasGRP1 expression in neuro-inflammation
Source: Sci Rep. 2020 Jul 1;10:10755. doi: 10.1038/s41598-020-67549-7 (PMC7329810; doi:10.1038/s41598-020-67549-7)

Supplementary Information

Full Title: **Nurr1 performs its anti-inflammatory function by regulating RasGRP1 expression in neuro-inflammation.**

Authors: Mihee Oh[1], Sun Young Kim[1], Jung-Eun Gil[1], Jeong-Su Byun[1], Dong-Wook Cha[1,4], Bonsu Ku[3], Woonghee Lee[5],Won-Kon Kim[2,4], Kyoung-Jin Oh[2,4], Eun-Woo Lee[2], Kwang-Hee Bae[2,4], Sang Chul Lee[2,4,*], and Baek-Soo Han[1,2,4,*]

Affiliations: [1]Biodefense Research Center, Korea Research Institute of Bioscience and Biotechnology, Daejeon 34141, Republic of Korea

[2]Metabolic Regulation Center, Korea Research Institute of Bioscience and Biotechnology, Daejeon 34141, Republic of Korea

[3]Disease Target Structure Research Center, Korea Research Institute of Bioscience and Biotechnology, Daejeon 34141, Republic of Korea

[4]Department of Functional Genomics, University of Science and Technology (UST) of Korea, Daejeon 34113, Republic of Korea

[5]MODNBIO Inc. Seoul 08378, Republic of Korea

Supplementary Figure S1.

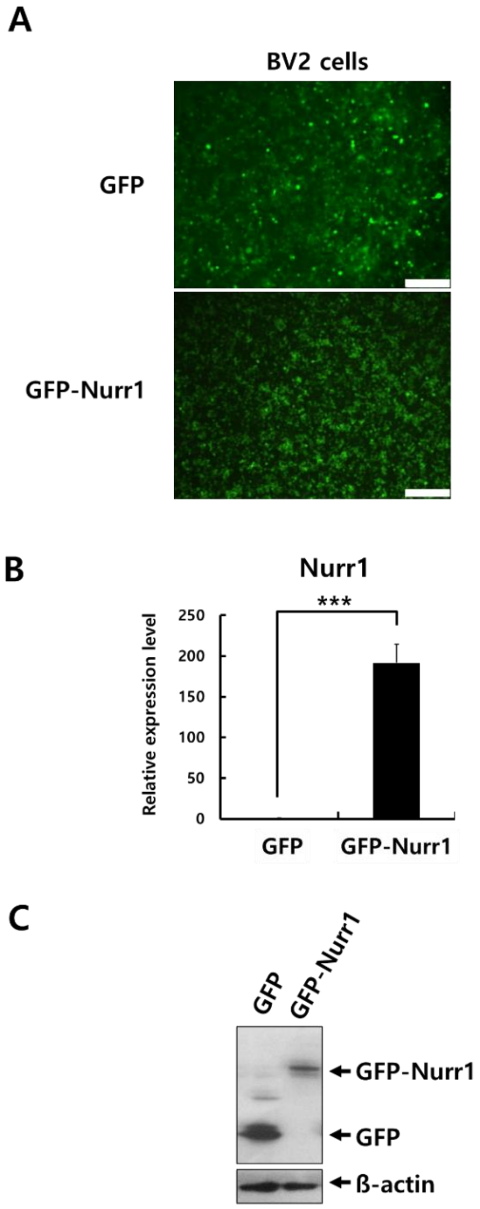

Supplementary Figure S1. Establishment of stable BV-2 cells expressing Nurr1.

(A) BV2 cells were infected with lentiviral particles containing GFP or GFP tagged Nurr1. After sorting with FACS, BV2 cells were maintained in culture medium containing puromycin (5 µg/ml). Images were obtained under a fluorescence microscope. Scale bars, 500 µm. (B and C) The expression levels of *Nurr1* mRNA (B) and protein (C) were analyzed by real-time PCR and western blot, respectively. Statistical analysis was performed using Student's t-test (means±SD; n=3; ***P<0.005).

Supplementary Figure S2.

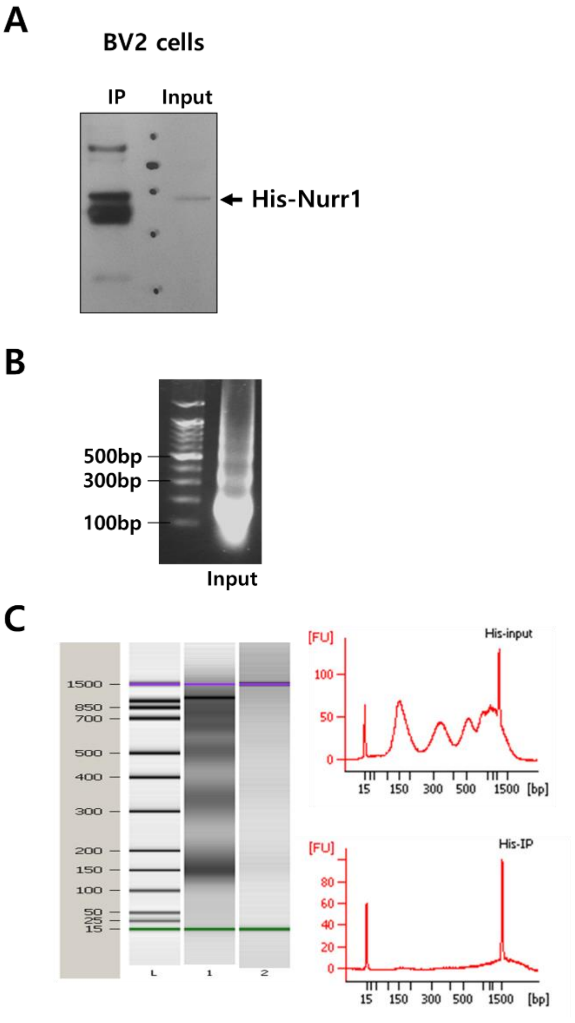

Supplementary Figure S2. The process for ChIP sequencing

(A) His tagged Nurr1 was transfected into BV2 cells and immunoprecipitated using anti-His antibody. (B) The fragmentation status of the chromatin samples was measured by DNA gel electrophoresis. (C) The concentration and quality of the immunoprecipitated protein-DNA complex were analyzed by DNA gel electrophoresis.

Supplementary Figure S3

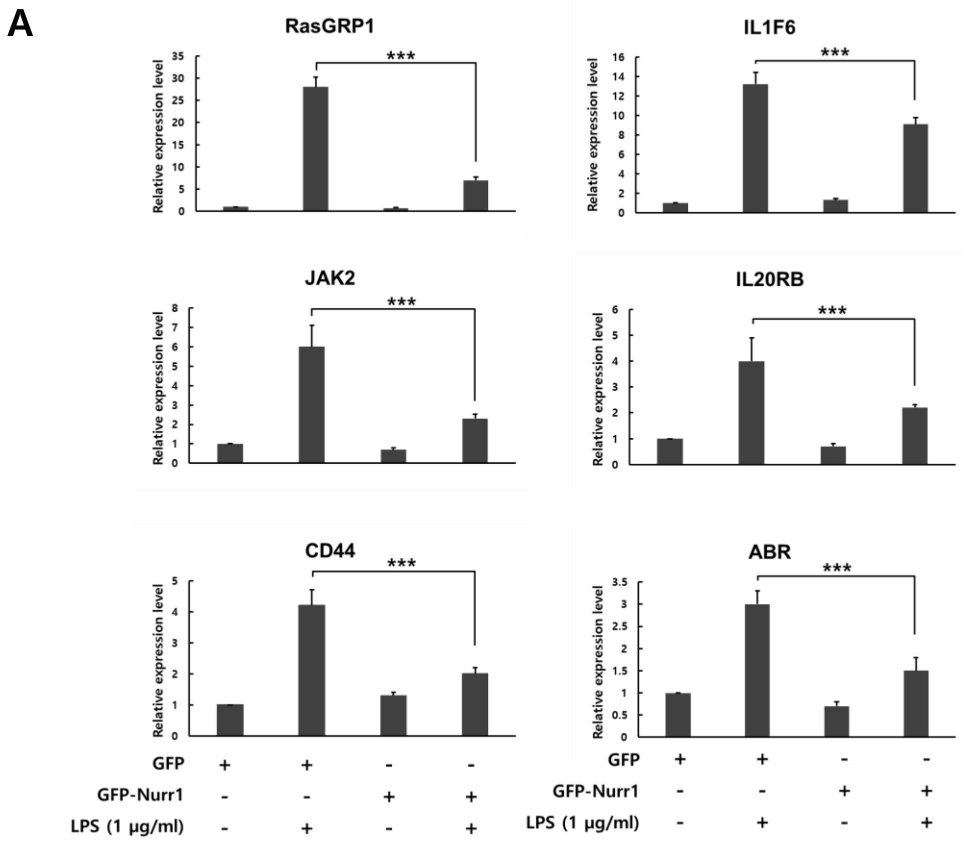

B

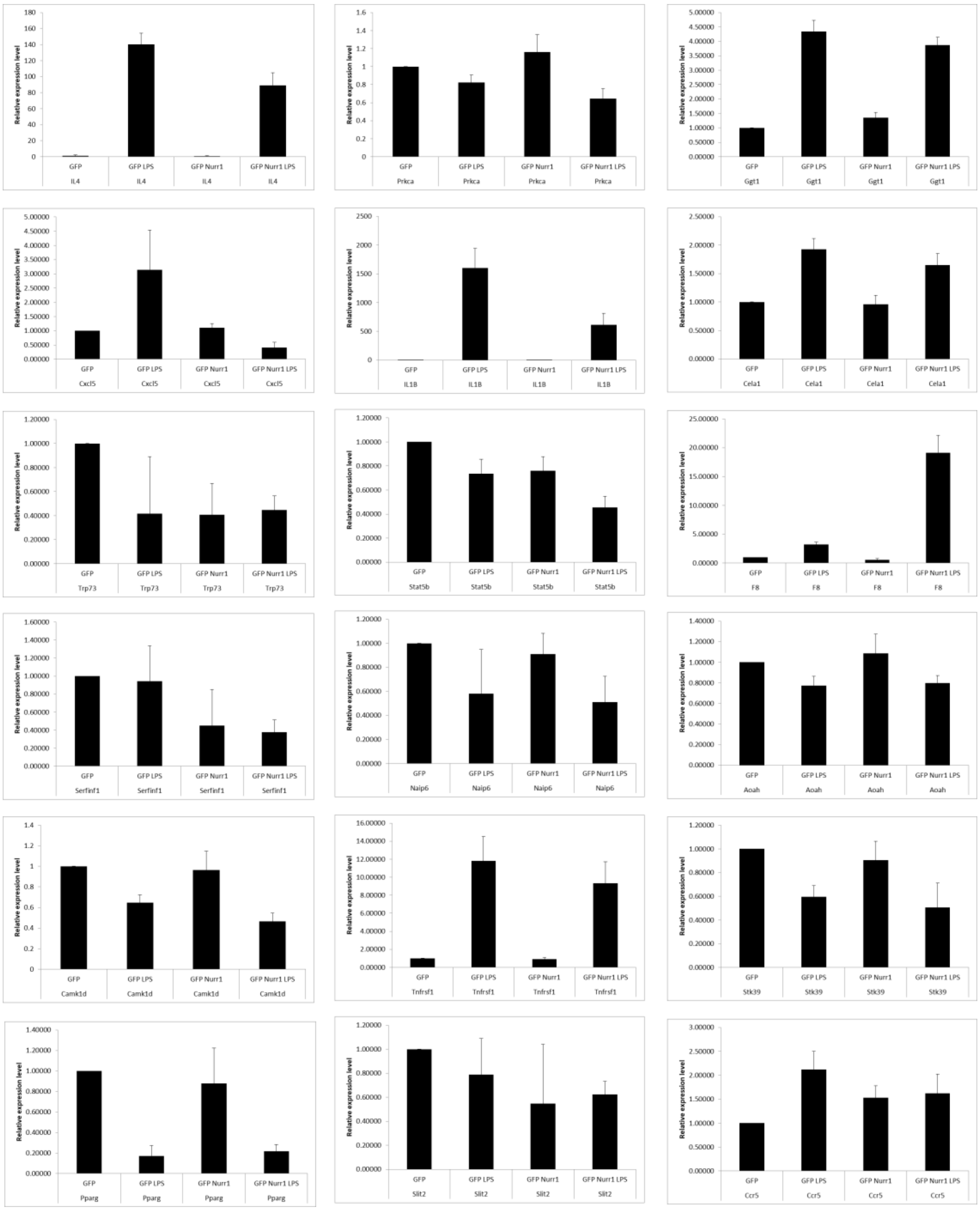
Relative expression level
GFP
GFP LPS
GFP Nurr1
GFP Nurr1 LPS
IL4
Prkca
Ggt1
Cxcl5
IL1B
Cela1
Trp73
Stat5b
F8
Serfinf1
Naip6
Aoah
Camk1d
Tnfrsf1
Stk39
Pparg
Slit2
Ccr5

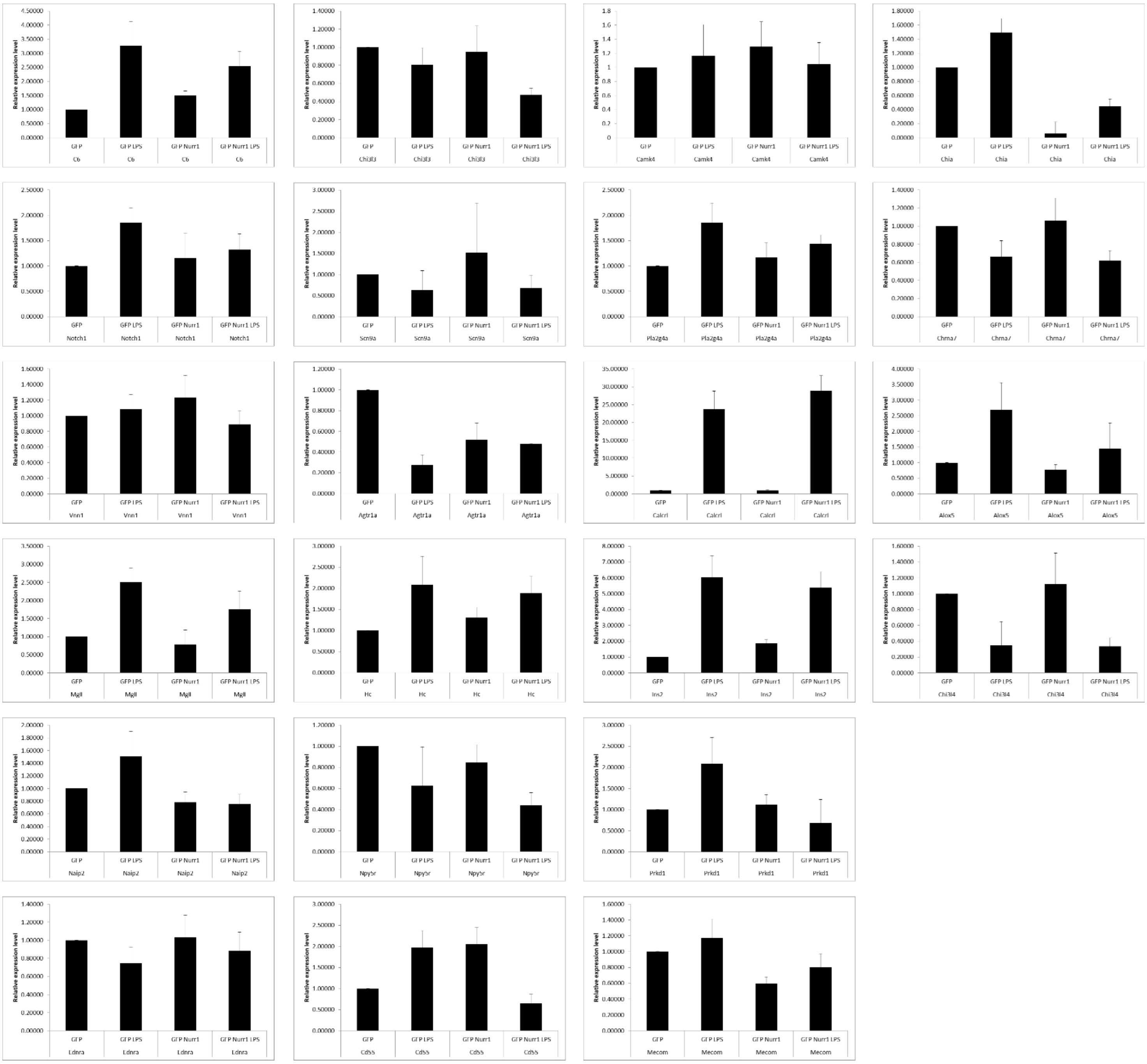

Supplementary Figure S3. Nurr1 down-regulated 6 genes containing RasGRP1 following LPS stimulation.

BV2 cells expressing GFP or GFP-tagged Nurr1 were treated with LPS (1 μg/ml) or saline for 6 h. The expression levels of mRNA were analyzed by a semi-quantitative real-time PCR and normalized with actin. These experiments were repeated three times. Statistical analysis was performed using Student's t-test (mean±SD; ***P<0.005).

Supplementary Figure S4.

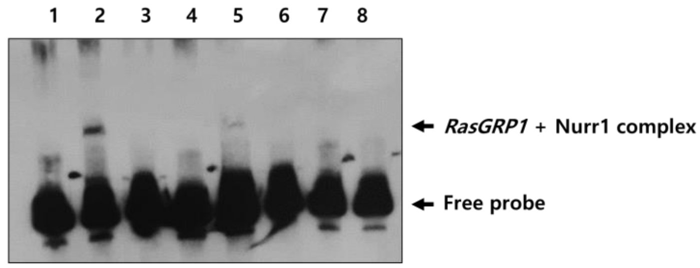

Lane 1 : GST
Lane 2 : GST-DBD
Lane 3 : GST-LBD
Lane 4 : GST
Lane 5 : GST-DBD
Lane 6 : GST-LBD
Lane 7 : probe alone
Lane 8 : probe alone

Supplementary Figure S4. Nurr1's LBD did not bind to specific sites in the second intron of the *RasGRP1* gene.

Biotin-labeled DNA probe (25 ng) with the NBRE-motif containing region in the second intron of the Rasgrp1 gene was incubated with purified recombinant GST, GST-DBD and GST-LBD (3.75 µg), and the DNA-protein complexes were separated on 6% native polyacrylamide gels. These experiments were repeated three times.

Uncropped blots in Figure 2.

Fig. 2.C

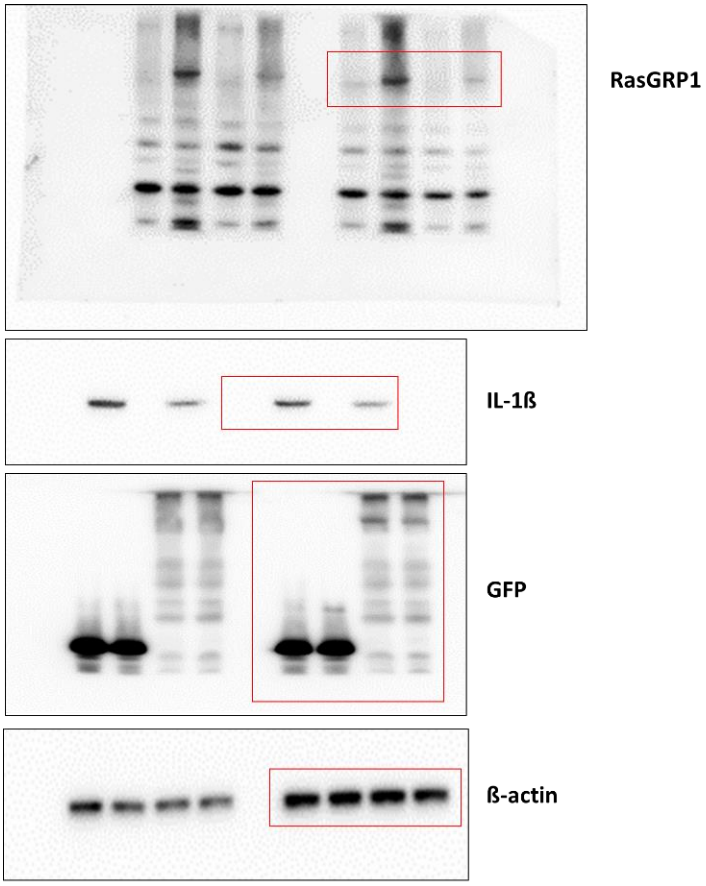

Fig. 2.E

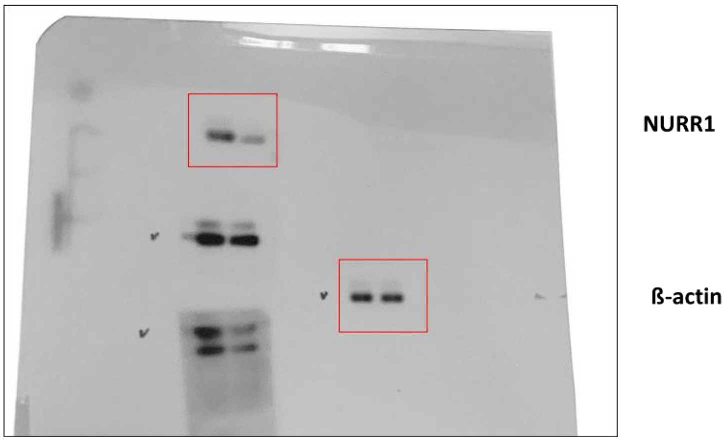

Uncropped blots in Figure 2.

Fig. 2.F

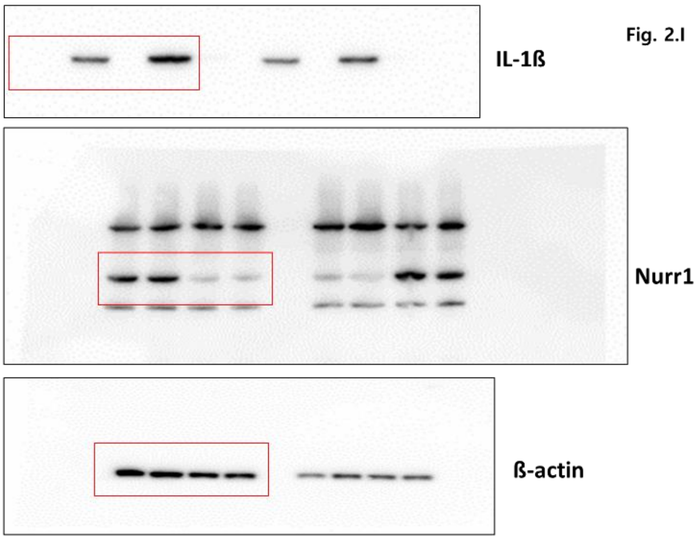

Fig. 2.I

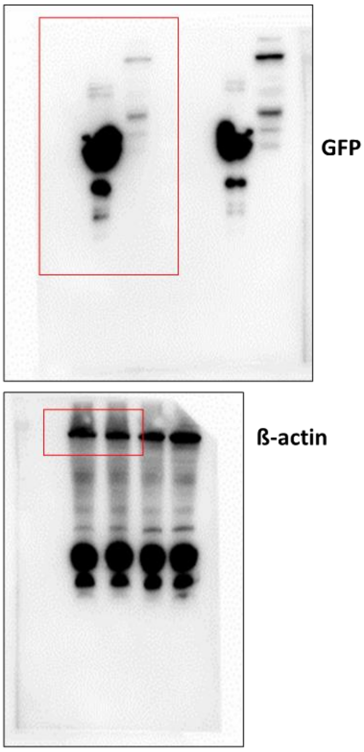

Fig. 2.K

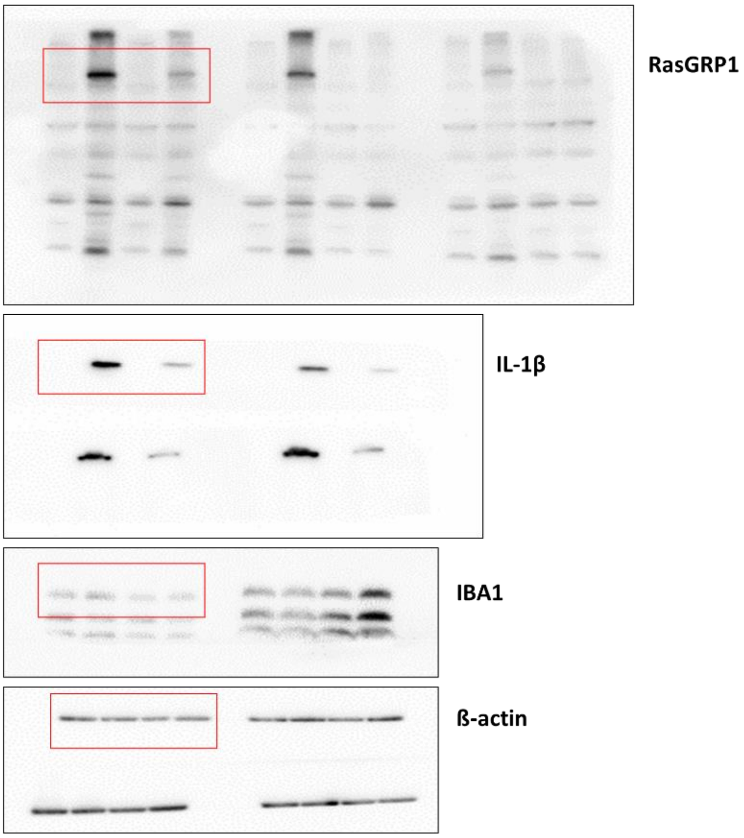

Uncropped blots in Figure 4.

Fig. 4.A

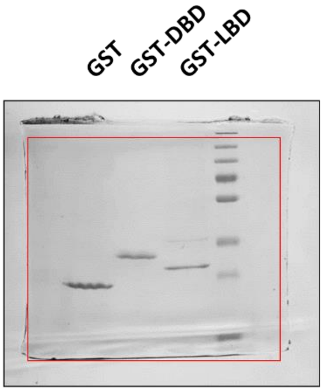

Fig. 4.B

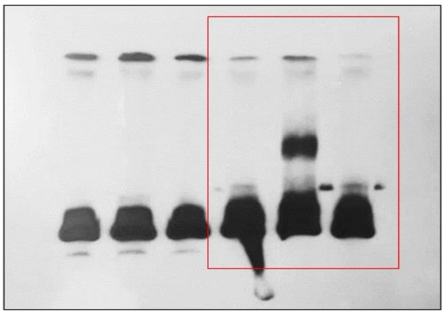

← *RasGRP1* + Nurr1 complex

← Free probe

Fig. 4.C

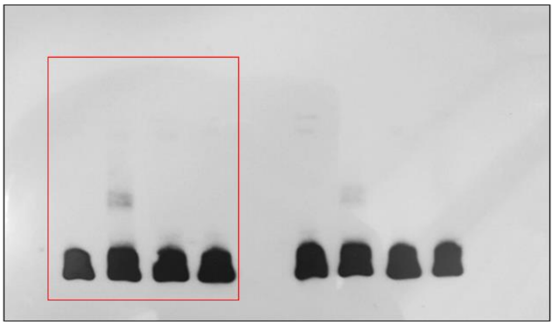

← *RasGRP1* + Nurr1 complex

← Free probe

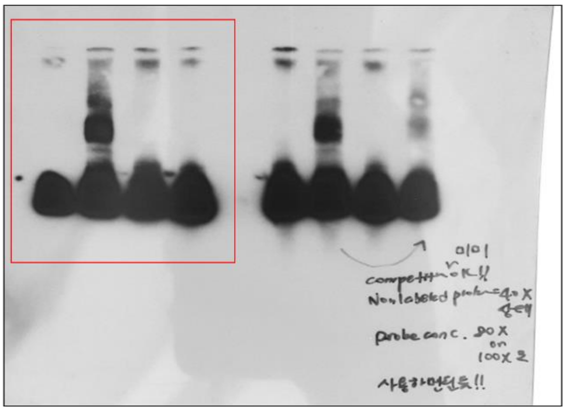

← *RasGRP1* + Nurr1 complex

← Free probe

Uncropped blots in Figure 6.

Fig. 6.B

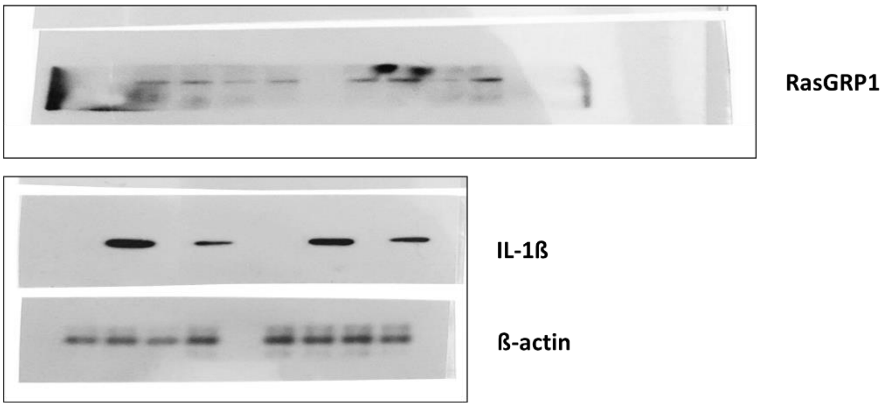

Uncropped blots in Figure 7.

Fig. 7.A

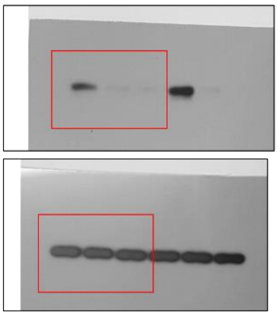

Fig. 7.B

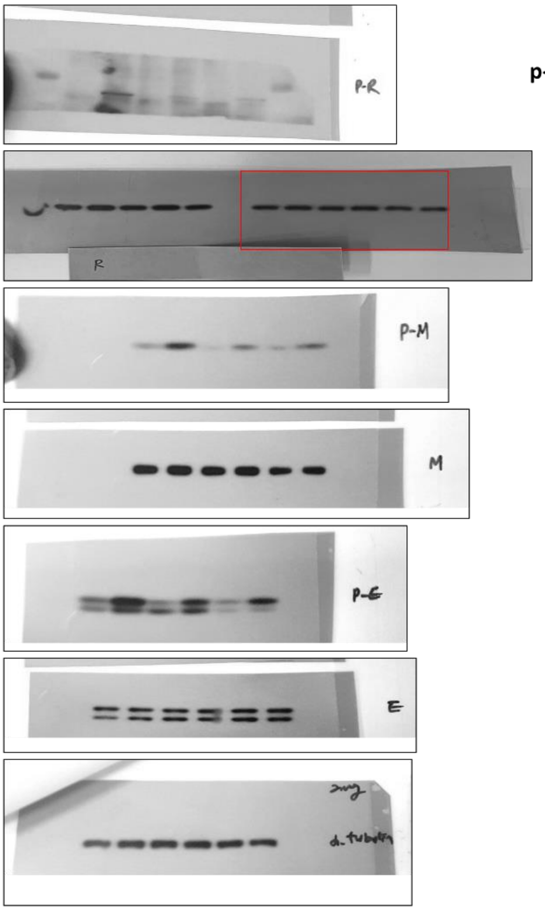

p-RAF

RAF

p-MEK

MEK

p-ERK

ERK

α-tubulin

Fig. 7.C

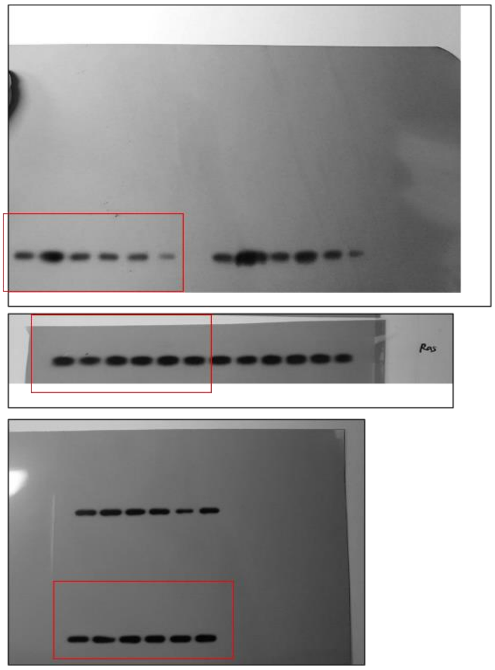

GTP-bound RAS

Total RAS

α-tubulin

Uncropped blots in Supplementary Figure S1.

Supplementary Fig.S1C

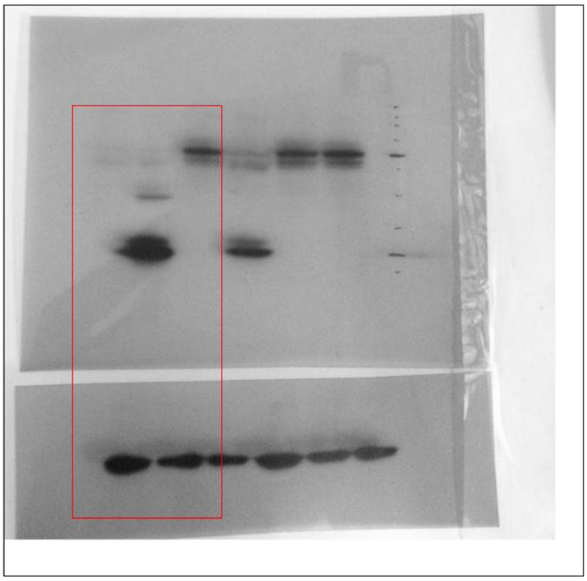

Uncropped blots in Supplementary Figure S2.

Supplementary Fig.S2A

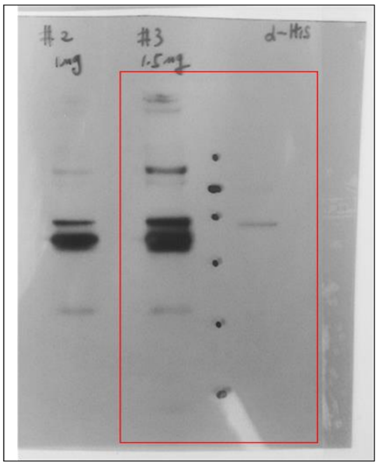

His-Nurr1

Uncropped blots in Supplementary Figure S3.

Supplementary Fig.S3

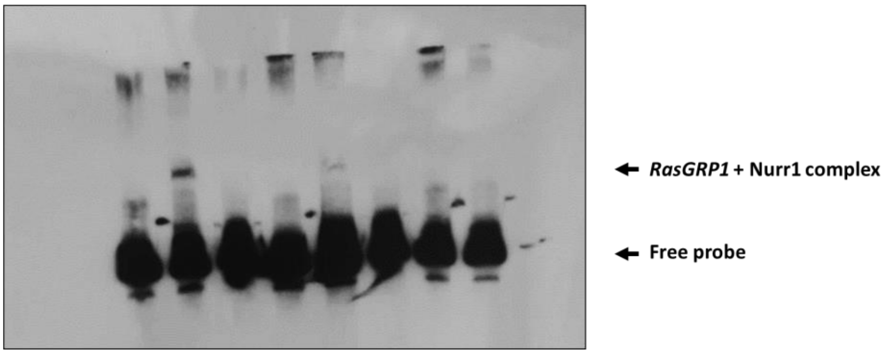

Supplement: Supplementary file 1 — Supplementary file1 (PDF 1699 kb) [file 41598_2020_67549_MOESM1_ESM.pdf]
